# Enhanced thermoelectric performance in polymorphic heavily Co-doped Cu_2_SnS_3_ through carrier compensation by Sb substitution

**DOI:** 10.1080/14686996.2021.1920821

**Published:** 2021-05-28

**Authors:** Yaqing Zhao, Yan Gu, Peng Zhang, Xiaohui Hu, Yifeng Wang, Peng’An Zong, Lin Pan, Yinong Lyu, Kunihito Koumoto

**Affiliations:** a College of Materials Science and Engineering, Nanjing Tech University, Nanjing, China; b State Key Laboratory of New Ceramics and Fine Processing, School of Materials Science and Engineering, Tsinghua University, Beijing, P.R.China; c Jiangsu Collaborative Innovation Center for Advanced Inorganic Function Composites, Nanjing Tech University, Nanjing, China; d Nagoya Industrial Science Research Institute, Nagoya, Japan; e Department of Research, Center of Nanotechnology, King Abdulaziz University, Jeddah, Saudi Arabia

**Keywords:** Co-doped Cu_2_SnS_3_, sb-substitution, carrier compensation, polymorphic, thermoelectric, 50 Energy Materials, 210 Thermoelectronics/Thermal transport/insulators

## Abstract

Heavily acceptor-doped Cu_2_SnS_3_ (CTS) shows promisingly large power factor (*PF*) due to its rather high electrical conductivity (*σ*) which causes a modest *ZT* with a high electronic thermal conductivity (*κ_e_*). In the present work, a strategy of carrier compensation through Sb-doping at the Sn site in Cu_2_Sn_0.8_Co_0.2_S_3_ was investigated, aiming at tailoring electrical and phonon transport properties simultaneously. Rietveld analysis suggested a complex polymorphic microstructure in which the cation-(semi)ordered tetragonal phase becomes dominant over the coherently bonded cation-disordered cubic phase, as is preliminarily revealed using TEM observation, upon Sb-doping and Sb would substitute Sn preferentially in the tetragonal structure. With increasing content of Sb, the *σ* was lowered and the Seebeck coefficient (*S*) was enhanced effectively, which gave rise to high *PF*s maintained at ~10.4 μWcm^−1^K^−2^ at 773 K together with an optimal reduction in *κ_e_* by 60–70% in the whole temperature range. The lattice thermal conductivity was effectively suppressed from 1.75 Wm^−1^K^−1^ to ~1.2 Wm^−1^K^−1^ at 323 K while maintained very low at 0.3–0.4 Wm^−1^K^−1^ at 773 K. As a result, a peak *ZT* of ~0.88 at 773 K has been achieved for Cu_2_Sn_0.74_Sb_0.06_Co_0.2_S_3_, which stands among the tops so far of the CTS-based diamond-like ternary sulfides. These findings demonstrate that polymorphic microstructures with cation-disordered interfaces as an approach to achieve effective phonon-blocking and low lattice thermal conductivity, of which further crystal chemistry, microstructural and electrical tailoring are possible by appropriate doping.

## Graphical Abstract

**Figure:**
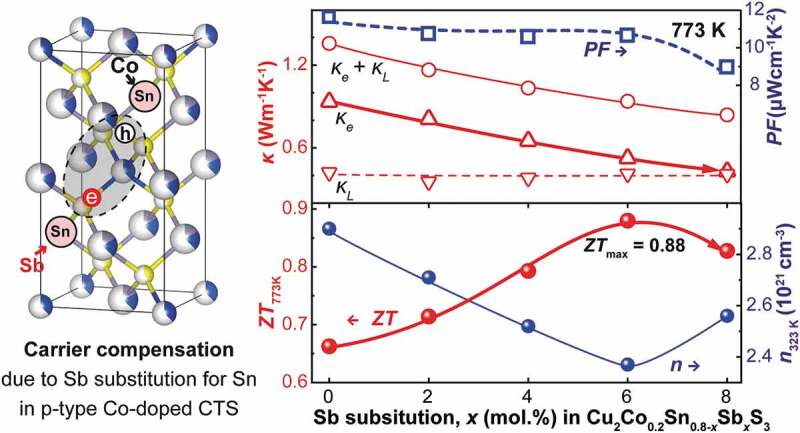

## 1. Introduction

Thermoelectric (TE) materials can convert a temperature gradient into an electrical voltage and vice versa, by utilizing the Seebeck effect and Peltier effect, respectively [1]. They can be used both in applications for thermal energy recovery from various heat resources [2] and for steady-state cooling for electronic devices [3,4], and therefore have been attracting wide attentions for decades [5]. The thermal-to-electric conversion efficiency for a TE material is determined by the dimensionless figure of merit, *ZT *=* S*^2^*σT*/(*κ_e_* + *κ_l_*) [6], where the *S, σ, T* are Seebeck coefficient, electrical conductivity, and the absolute temperature, respectively, and the *κ_e_* and *κ_l_* are the electronic and the lattice components, of which the sum represents the total thermal conductivity, *κ*, for a TE material. In particular, from the viewpoint of scalable commercial application, intensive efforts have been made in research of TE materials beyond the currently most-widely used heavy alloys, such as compounds of Te, Bi, Pb and etc., most of which are naturally scarce and/or toxic. Thus, a multitude of environmentally friendly thermoelectric sulphides has been reported, such as chalcopyrite CuFeS_2_ [7,8], tetrahedrite [9,10], paracostibite CoSbS [11,12] and thiospinels [13–15].

Mohite-type diamond-like ternary compound of Cu_2_SnS_3_ (hereafter CTS) has emerged as a new environmental-friendly candidate in recent years due to its phonon-glass-electron-crystal characteristics [16–18] among the high-performance TE sulfides such as synthetic colusites [19,20]. Structurally, phase transition after doping is common and has already been reported in several references [21–25], and in our samples, it adapts three different variants including monoclinic, cubic and tetragonal phases (hereafter referred to as m-CTS (*a* = 6.653 Å, *b* = 11.537 Å, *c* = 6.665 Å [26]), c-CTS (*a* = *b* = *c* = 5.43 Å) and t-CTS (*a* = *b* = 5.413 Å, *c* = 10.824 Å [27]), respectively) with the space group of *Cc, F*-43 *m* and *I*-42, respectively. According to the theoretical work by Zhang et al. [16], m-CTS is a direct-gap semiconductor containing three bands deriving from the strong hybridization between Cu-3*d* and S-3*p* orbitals in the valence band edge and one single band from Sn-4*s* in the conduction band (CB) edge, which means clearly the priority for p-type TE performance with a 3D hole transport channel mainly consisting of Cu-S and S-S networks, and moreover, the benefit for carrier concentration optimization by alloying and/or doping at the Sn site that can help suppress *κ_l_* while deteriorating little the electrical conduction. Meanwhile, it has a very low intrinsic *κ_l_* at high temperatures which approaches its theoretical minimum of about 0.3 W m^−1^ K^−1^ at 773 K, suggesting a promising prospective for mid-temperature TE applications.

Indeed, experimental research indicated that heavily acceptor-doping using transition metals, such as Zn [17], Ni [28], Mn [21], Fe [18], Cu [29], In [30] etc., can cause a structural transformation from cation-order low-symmetry (m-CTS) to disorder high-symmetry (t-CTS and c-CTS) phases which not only helps to reach an ultralow *κ_l_* due to the strengthened anharmonicity, but also optimize the transport properties through band engineering and the enhanced carrier concentration, synergistically giving rise to high power factors (*PF *=* S^2^σ*) of ~8.0 μW cm^−1^ K^−2^ and maximal *ZT* values of about 0.6–0.7 around 773 K. Moreover, a similar result can even be obtained in the recent report via ab initio calculation without resorting to chemical alloying [31]. Particularly, acceptors with unfilled *d*-orbitals (e.g. Co [32] and Mn [19]) are found to be able to enhance the carrier DOS (density-of-states) effective mass m*, possibly due to the participation of their *d*-orbitals in the upper valence bands, resulting in even higher *PFs* of 9.0 ~ 11.0 μW cm^−1^ K^−2^ at high temperatures and a peak *ZT* of 0.85 at 723 K in 20%Co-doped CTS (Cu_2_Sn_0.8_Co_0.2_S_3_).

However, heavy doping for CTS usually causes a highly degenerate state with a large *σ*, up to 1500 S cm^−1^ around 300 K, and consequently a considerable *κ_e_* up to 0.9–1.0 W m^−1^ K^−1^, which accounts for over ~2/3 of the total *κ* and limits the *ZT* noticeably. Thus, it should be a feasible way to enhance the TE performance by properly lowering the *σ* while maintaining the high *PF*.

In the present work, an approach of carrier compensation through donor-doping at Sn site using Sb is put forward for Cu_2_Sn_0.8_Co_0.2_S_3_ to examine the effect in tailoring the electrical transport properties as mentioned above, in view of that the Sb substitution for Sn can generate electrons which would help reduce the high hole carrier concentration by electron-hole recombination while affecting little the conducting states, and in addition, suppress the lattice thermal conductivity due to the high-entropy effect.

## 2. Experiment

Powder samples with a nominal composition of Cu_2_Sn_0.8−*x*_Sb*_x_*Co_0.2_S_3_ (*x* = 0, 0.02, 0.04, 0.06, 0.08) were prepared by high temperature reaction. First, high purity element powders of Cu, Sn, Sb, Co and S were weighed and mixed thoroughly in a mole ratio of 2: 0.8-*x: x*: 0.2: 3. The mixtures were sealed under high vacuum in silica tubes and heated in a box furnace at a rate of 5 K min^−1^ to 1193 K, held there for 8 h, then rapidly cooled to 983 K and kept for 48 h, finally the mixtures were cooled down naturally to room temperature to obtain the compound ingots. The synthesized ingots were thoroughly ground into powders in an Al_2_O_3_ mortar, and then sieved. The powders were sintered into compact pellets (over 95% theoretical density) in graphite dies at 823 K for 5 min under an axial pressure of 50 MPa and vacuum (< 5 Pa) by spark plasma sintering (SPS).

The phase composition and crystal structure of the samples were checked by X-ray diffraction (XRD) analysis with an ARL X’TRA diffractometer (SmartLab3, RIGAKU, Japan) using Cu Kα radiation. Sample’s morphology was observed by field-emission scanning electron microscopy (FE-SEM, FEI Nova NanoSEM450). The microstructure was conducted using a transmission electron microscopy (JEM-2100 F). The Seebeck coefficients and electrical conductivities were measured in the radial direction of a bar-shaped specimen with dimensions of 10 mm × 2 mm × 3 mm by a conventional steady state method and a four-probe method, respectively, in a He atmosphere at 323 to 723 K with a commercial system (LSR-3, Linseis). X-ray photoelectron spectroscopy (XPS) was performed with a Physical Electronics system (ESCALAB 250Xi) using a standard Al Kα source. Thermal conductivity was calculated by *ĸ = DρC_p_*, where the *D* was thermal diffusivity measured in the axial direction of a disk-shaped sample of Φ 10 mm × 1 mm using a Netzsch laser flash diffusivity instrument (LFA457, Netzsch, Germany), the *C_p_* was the specific heat capacity calculated according to the Dulong–Petit law [33], and *ρ* was the mass density measured using the Archimedes method. Hall effect measurements for the carrier concentration and Hall mobility were conducted with a van der Pauw configuration under vacuum using a ResiTest8300 system (Toyo Tech. Co., Japan). The uncertainties of *S* and *σ* measurements are ±3 and ±5%, respectively, leading to a *PF* error of ±10%. Combining with the uncertainty of ±10% for *κ* measurements, the error bar of *ZT* is estimated to be no more than ±20%.

## 3. Results and discussion

**Figure 1.**
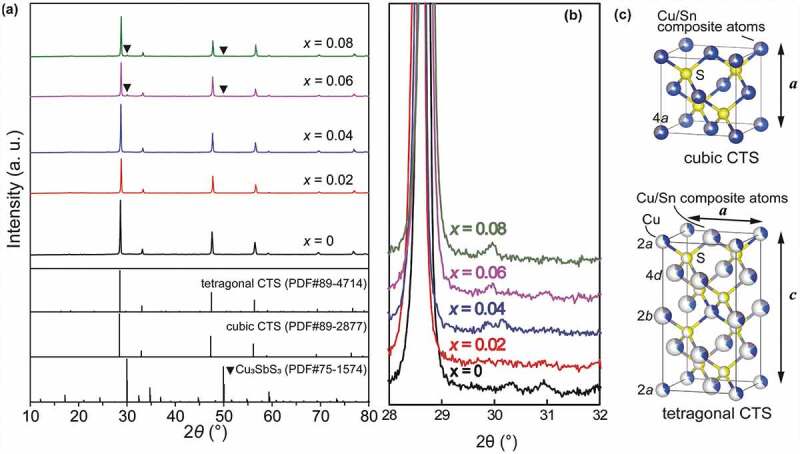
XRD patterns of (a) the Cu_2_Sn_0.8-*x*_Sb*_x_*Co_0.2_S_3_ (*x* = 0, 0.02, 0.04, 0.06 and 0.08) bulk samples and (b) the enlarged area from 29° to 31° in (a), together with the crystal structure illustrations of the (c) cubic and tetragonal phases

Figure 1(a) shows XRD patterns for all the Cu_2_Sn_0.8-*x*_Sb*_x_*Co_0.2_S_3_ (*x* = 0, 0.02, 0.04, 0.06 and 0.08) bulk samples, together with illustrations of crystal structures for the cubic and tetragonal phases (Figure 1(b,c)). All the samples, as can be seen, are basically composed of t-CTS and/or c-CTS phases when *x* is less than 0.06. With *x* ≥ 0.06, peaks (222) and (440) for Cu_3_SbS_3_ (PDF#27-1745) at 30° and 50° are found. In the c-CTS, the cations are completely disordered at the 4*a* sites, statistically of 2/3 Cu and 1/3 Sn, and in the t-CTS, the 2*a* sites are occupied by 3/8 Cu and 5/8 vacancy, and the 2*b* and 4*d* sites by composite atoms of M_1_[43.6%Sn+56.4%Cu] and M_2_[46.3%Sn+53.7%Cu], respectively. This means that in these doped CTS, the cations including Cu, Sn, and Co and Sb are semi-ordered in t-CTS or fully disordered in c-CTS, which contributes significantly to achieve the very low *κ_l_*. Moreover, the improved symmetry of the S-centered SM_4_ (M = metal atoms) tetrahedra compared to that in the m-CTS structure should be also beneficial to give an enhanced degeneracy of the orbitals in the upper VB and thus an increase of DOS near the Fermi level.

**Figure 2.**
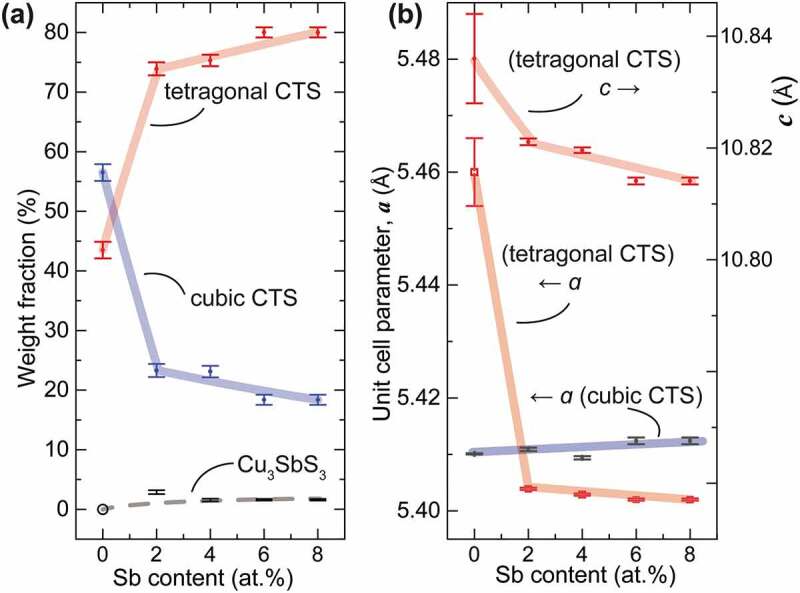
(a) The weight fractions of the c-CTS, t-CTS and Cu_3_SbS_3_ phases, and (b) the lattice parameters of *a* and *c* of the two CTS phases, derived from Rietveld refinement of the XRD data. Note that in the refinement the nominal composition of Cu_2_SnS_3_ were used for simplicity

To analyse the change of phase composition and crystal structure due to Sb doping, Rietveld refinement was conducted using a BGMN-cored program Profex [34] with these 3 phases taken into account, which adopted a polymorphic model consisting of c-CTS, t-CTS and m-CTS phases and ended with a low *R_wp_* of 3.1–4.3% (in Table 1) suggesting high goodness-of-fitting. (see Figure S1 for the Rietveld refinement result of *x* = 0.06 sample) As summarized in Figure 2(a), the weight fraction of t-CTS increased abruptly from about 45 wt.% to about 75% in the *x* = 0.02 sample, then gradually to 80% at *x* = 0.08. Correspondingly, the fraction of the c-CTS phase dropped largely from about 55 to 22% with *x* = 0.02, then gradually to about 18% with *x* increasing to 0.08. Meanwhile, the amount of impurity Cu_3_SbS_3_ was kept at a very low level of ~1.6%. This result indicates t-CTS phase was much favored upon Sb substitution. Moreover, as shown in Figure 2(b), the unit cell parameter, *a*, increased very little, while *a* and *c* contracted drastically with 0.02 at% Sb incorporation, and were further reduced with increasing *x*. This agrees well with relatively smaller ionic radius of Sb^5+^ (60 pm for coordination number C.N. = 6) than that of Sn^4+^ (69 pm for C.N. = 6), and suggests strongly that the Sb substitution should take place predominantly in the t-CTS phase. More interestingly, this signifies that Sb-doping might have caused a remarkable change in the coordination configuration of SM_4_ tetrahedra in t-CTS phase.

**Table 1.** The weight fractions of the c-CTS, t-CTS and Cu_3_SbS_3_ phases, the lattice parameters of *a, b* and *c* of the two CTS phases, and the *R_wp_* derived from Rietveld refinement of the XRD data

*x*	Weight fraction	*a*(Å)	*b*(Å)	*c*(Å)	*R_wp_*(%)
0	c-CTS (56.5%)	5.4101	5.4101	5.4101	4.28
	t-CTS (43.5%)	5.46	5.46	10.83	
0.02	c-CTS (23.2%)	5.4112	5.4112	5.4112	3.61
	t-CTS (73.95%)	5.4038	5.4038	10.8209	
	Cu_3_SbS_3_(2.85%)				
0.04	c-CTS (23%)	5.4094	5.4094	5.4094	3.22
	t-CTS (75.43%)	5.4032	5.4032	10.8196	
	Cu_3_SbS_3_(1.57%)				
0.06	c-CTS (20.05%)	5.4124	5.4124	5.4124	3.1
	t-CTS (78.36%)	5.4025	5.4025	10.8196	
	Cu_3_SbS_3_(1.59%)				
0.08	c-CTS (18.81%)	5.4104	5.4104	5.4104	3.11
	t-CTS (79.62%)	5.4020	5.4020	10.8141	
	Cu_3_SbS_3_(1.57%)

**Figure 3.**
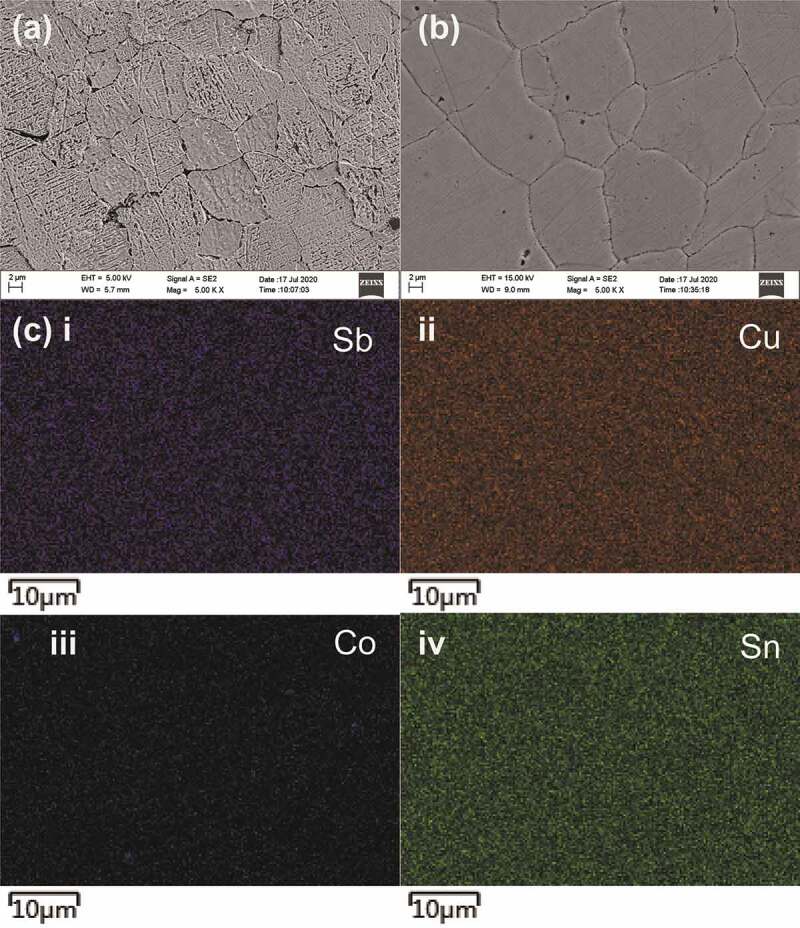
FE-SEM images for the *x* = 0 (a) and *x* = 0.08 sample’s thermally-etched mirror-polished surfaces, and the EDX mappings for the constituent elements (c i–iv) for the *x* = 0.08 sample

However, we didn’t find any obvious inhomogeneity of elemental distribution by SEM and energy-dispersive X-ray spectroscopy (EDX). As shown in Figure 3(a and b) for the SEM images for thermally etched mirror surface of the merely Co-doped CTS and the *x* = 0.08 sample, respectively, no remarkable difference in grain growth has been found due to the Sb substitution. EDX mappings revealed homogeneous distribution of all elements, without preferential Sb segregation in the grains or to the grain boundaries, which suggests that the c- and t-CTS might have formed a very special microstructure that is possibly featured with nanoscale intergrowth or even more complicated nano-texture, which should resemble the one that we have revealed in Zn-doped CTS previously [14].

**Figure 4.**
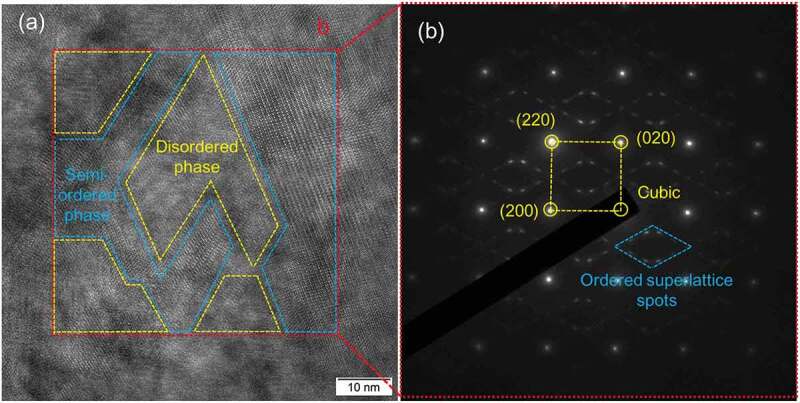
(a) HRTEM images for the *x* = 0.06 sample, where the mosaic structure is consisted of disordered (yellow) and semi-ordered (blue) phases; (b) SAED patterns for the red region of (a), showing the main spots forming the yellow square and the weak spots, corresponding to the zinc-blende-type disordered region and the semi-ordered superstructure [35]

In order to verify this special microstructure, transmission electron microscopy (TEM) observation was conducted for the *x* = 0.06 sample and a typical TEM image is shown in Figure 4. Figure 4(a) shows, a mosaic-type domain nanostructure in the matrix, comprising approximately 10 nm wide faceted domains with cation columns of a uniform contrast (see the yellow frames) that are coherently bonded to a surrounding network phase with a distinctly different periodic contrast (see the blue frames), corresponding to a cation-disordered zinc-blende-like phase and a semi-disorder one, respectively [35]. This special configuration represents a new kind of 3-dimensional phonon-glass electron-crystal nanostructure, since the cation-disordered domains and the abrupt domain walls are very effective in phonon-blocking like that in Cu-Zn-Sn-S quaternary system [35]. Moreover, the selected area electron diffraction (SAED) in the red square region shown in Figure 4(b) exhibits the main diffraction spots (see the yellow square corners) and weak split superlattice diffraction spots (in the blue diamond frame), deriving from the zinc-blende phase and the semi-ordered phase, respectively, like that of Cu_2_Zn_0.15_Sn_0.85_S_3_ [36]. Interestingly and particularly, the cation ordering or disordering plays a significant role in the phonon and electron transport properties, e.g. in the Zn-doped CTS [36], a higher conductivity in the semi-ordered phase than in the ordered one has been found, and similarly, a high carrier mobility of 11.7 cm^2^V^−1^s^−1^ at 300 K has been reported in a monoclinic ordered phase of Cu_5_SnS_7_ even with a high concentration of 5.75 × 10^21^ cm^−3^ [37]. Therefore, optimization of the hierarchical architecture of these materials, e.g. by intentional selective tailoring of the crystal chemical composition and thus the transport properties of the different phases, would represent a novel strategy for achieving high performance in the CTS-based materials [17].

**Figure 5.**
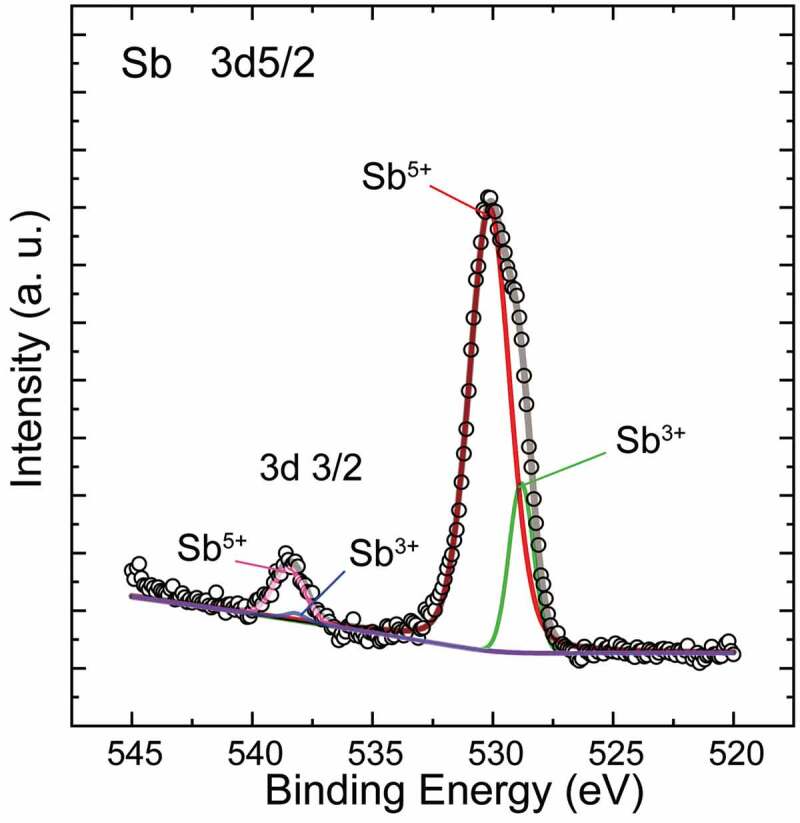
XPS profile for the *x* = 0.04 sample, where the main peak can be decomposed into profiles for the co-existing 5+ and 3+ states. The much stronger intensity of the former indicates the 5+ state should be dominant as a whole

To clarify the oxidation state of Sb in the samples, XPS data was collected typically for *x* = 0.04 and is plotted within a local binding energy range for Sb 3*d*3/2 and 3*d*5/2 in Figure 5. According to the peak processing, the asymmetric main peak is composed of the characteristic profiles for both Sb^5+^ (at 530.14 and 538.48 eV) and Sb^3+^ (at 528.82 and 538.2 eV). In addition, the former’s intensity is much stronger than the latter’s, indicating a higher proportion of 5+ states than the co-existing 3+ states. Based on charge balance, it is safe to assume the signal of Sb^3+^ should be from the Cu_3_SbS_3_ phase as an impurity. However, the appearance of weak Sb^3+^ signal, despite the absence of Cu_3_SbS_3_ peak in XRD pattern in the *x* = 0.04 sample, suggests that the Sb doped into the CTS phase should be in a mixed state where Sb^5+^ should be dominant relative to Sb^3+^. Therefore, it would be reasonable to deduce that the Sb should have acted as a donor when substituting the Sn^4+^ sites or Cu^+^ sites, which can be also supported by the electrical transport properties as follows.

**Figure 6.**
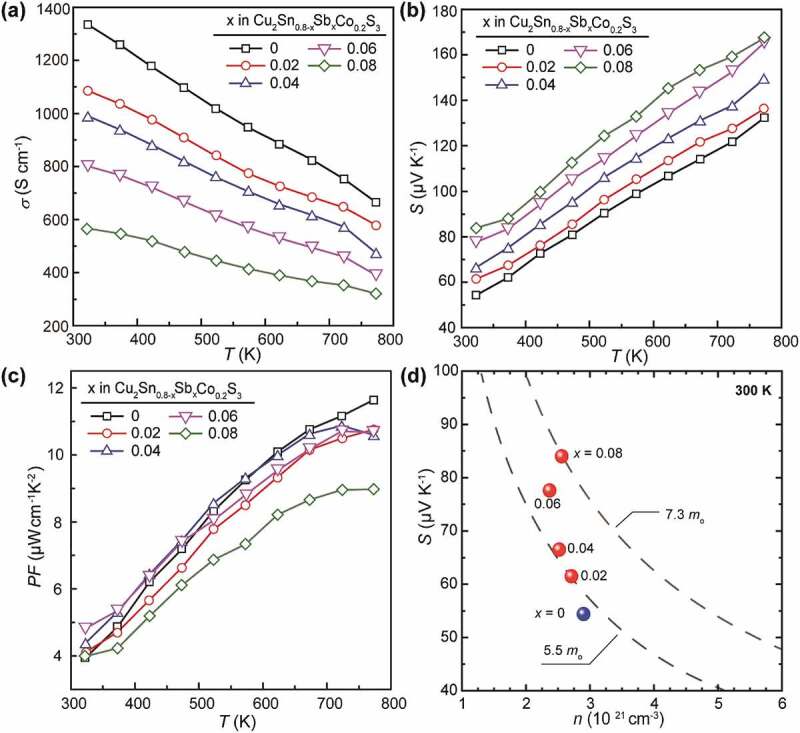
Thermoelectric properties of (a) electrical conductivity, (b) Seebeck coefficient and (c) power factor for the Cu_2_Sn_0.8-*x*_Sb*_x_*Co_0.2_S_3_ (*x* = 0, 0.02, 0.04, 0.06 and 0.08) samples as a function of temperature. (d) the Pisarenko plot of *S* vs *n* at 300 K for the samples

As one can see in Figure 6(a), the *σ* values for the Cu_2_Sn_0.8-*x*_Sb*_x_*Co_0.2_S_3_ (*x* = 0, 0.02, 0.04, 0.06 and 0.08) samples all decrease as temperature rises, and the slope of the log *σ* vs log *T* relation is about −1 for all. Since the carrier concentration should be almost constant in such highly degenerate CTS materials, as has been verified in the Zn-doped CTS [17], this means a same temperature dependence of carrier mobility and the carriers are predominantly scattered by acoustic phonons in all sample. Moreover, the *σ* for the Sb-substituted samples drops effectively with increasing *x*, from about 1320 S cm^−1^ for the merely Co-doped one to ~560 S cm^−1^ for the *x* = 0.08 one at 323 K, and the large discrepancy is even retained at 773 K. To verify the reason for the strong reduction of *σ* upon Sb substitution, Hall effect measurement for the carrier concentration *n* and Hall mobility *µ*_Hall_ was conducted. Results (in Table 2) showed a stepped reduction of *n* by ~1.7 × 10^20^ cm^−3^ corresponding to every increase of 0.02 at% Sb with *x* = 0 ~ 0.06, which is close to the estimated electron density, ~1.3 × 10^20^ cm^−3^, based on the assumption of fully ionized Sb^5+^ substituting for Sn^4+^, and the larger reduction in the determined *n* is possibly caused by the substitution of Sb for other atoms (Cu and Co), which would create more electrons to compensate for holes. The unexpected increase of *n* with *x* increasing from 0.06 to 0.08 is considered to be caused possibly by the increased formation of Cu vacancies. A possible reason may be linked with the effect of the secondary phase Cu_3_SbS_3_, since, according to the chemical composition, the formation of one Cu_3_SbS_3_ molecule always companies the generation of a Cu vacancy in Cu_2_SnS_3_, which can cause an increase of carrier concentration and a decrease in carrier mobility due to impurity scattering at the same time.

**Table 2.** Carrier concentration *n*, mobility *µ*_Hall_ determined by Hall effect measurements and effective mass of carriers m* at 300 K estimated based on SPB model for Cu_2_Sn_0.8-*x*_Sb*_x_*Co_0.2_S_3_ (*x* = 0, 0.02, 0.04, 0.06 and 0.08) samples

*x*	0	0.02	0.04	0.06	0.08
*n* (10^21^ cm^−3^)	2.90	2.71	2.52	2.37	2.56
*µ* (cm^2^V^−1^s^−1^)	2.87	2.50	2.45	2.11	1.38
m* (*m*_o_)	5.11	5.53	5.69	6.38	7.27

Besides, the mobility *µ*_Hall_ is comparable to that in Zn [24] or In-doped CTS [30], and decreases abnormally with decreasing *n* as Sb content increases. This indicates the intensified carrier scattering due to factors other than carrier interactions, which could be related with the decreasing fraction of c-CTS that has a higher *σ* than t-CTS [35].

Accordingly, the *S* values are enhanced as a result of the reduced *n* due to Sb doping, from ~54 μV K^−1^ for the *x* = 0 sample at 323 K to ~84 μV K^−1^ for the *x* = 0.08 one (see Figure 6(b)). The steady increase of *S* with temperature suggests no bi-polaron effect has occurred and the compensation for holes by electrons due to Sb doping is stable in the present temperature range. In order to clarify the mechanism for the enhancement of *S*, a Pisarenko plot (i.e. *S* vs *n*) for all the Cu_2_Sn_0.8-*x*_Sb*_x_*Co_0.2_S_3_ (*x* = 0, 0.02, 0.04, 0.06 and 0.08) samples was demonstrated in Figure 6(d), where 2 dashed lines with constant carrier effective mass, m*, estimated according to the single parabolic band (SPB) model relation of $S=\frac{8\pi^{2}k_{b}^{2}}{3eh^{2}}m^{*}T{\left(\frac{\pi }{3n}\right)}^{2/3}$ for the case of carriers being scattered dominantly by acoustic phonons [38]. As can be seen, the m* increased gradually with increasing amount of Sb from ~5.5 *m_o_* to ~7.3 *m_o_*. As reported previously, a larger *n* is usually accompanied with the decrease in the Fermi level down in the VB, and this would cause an increase in m* due to the involvement of non-converged sub-bands located at deeper energy levels, even causing invalidity of SPB model in CTS-like compounds with complex band structures for deviation from parabolicity of the bands near the Fermi level. It should be noted that the Sb doping has caused the reduction of *n*, i.e. the elevation of Fermi level, which should have brought about a decrease rather than an increase in m*. However, the experimental data showed an opposite trend. A possible reason for this anomaly might be related to the electronic contribution from Sb 5*s* and 5*p* states with the Cu-3*d* orbitals in the valence bands. Actually, a similar effect has been clarified in CuSbS_2_ [39], which would cause a net increase of density-of-states (DOS) near the Fermi level when the lone pair electrons first interact with S 3*p* electrons and the full antibonding orbital of this interaction is then sufficiently high so that it can subsequently interact with empty Sb 5*p* states, resulting in bonding states in the valence band and antibonding states at the bottom of the conduction band.

As a result, the power factors (*PF = S^2^σ*) in these Cu_2_Sn_0.8-*x*_Sb*_x_*Co_0.2_S_3_ (*x* = 0, 0.02, 0.04, 0.06 and 0.08) samples are almost maintained, as shown in Figure 5(c), except for a slight drop at 773 K. It should be noted that these *PFs* (~10.4 μW cm^−1^ K^−2^ at 723 ~ 773 K) are still quite high as compared with that reported in the literature [19,24,25,28–30] so far for Cu_2_SnS_3_-based materials, and moreover, they are achieved by effective reduction of *σ* and simultaneous enhancement of *S*, which greatly suppresses the electronic thermal conductivity *κ_e_* and consequently the total *κ*.

**Figure 7.**
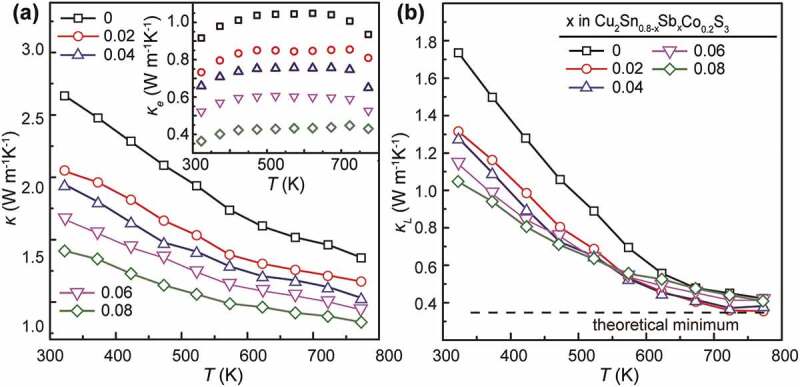
Thermal conductivities (*κ, κ_e_* and *κ_l_*) for all the Cu_2_Sn_0.8-*x*_Sb*_x_*Co_0.2_S_3_ (*x* = 0, 0.02, 0.04, 0.06 and 0.08) samples. The dash line in (b) indicates a theoretical minimum of *κ_l_* = 0.3 W m^−1^ K^−1^ at high temperatures

Figure 7 shows the thermal conductivities (*κ, κ_e_* and *κ_l_*) for Cu_2_Sn_0.8-*x*_Sb*_x_*Co_0.2_S_3_ (*x* = 0, 0.02, 0.04, 0.06 and 0.08) samples. One can see in Figure 7(a) that the total *κ* for the *x* = 0 sample falls in a high range between 2.7 W m^−1^ K^−1^ and 1.7 W m^−1^ K^−1^ at 323 and 773 K, respectively. With an increase in Sb amount, the *κ* value is reduced significantly to 1.7 W m^−1^ K^−1^ and 0.88 W m^−1^ K^−1^. In the total *κ*, the *κ_e_* values as a component are calculated from the relation, *κ_e_* = *LσT*, where *L* is the Lorenz number estimated using the formula *L* = 0.5 + exp(*S*/116) [40]. Thanks to the large reduction in *σ*, the *κ_e_* values, as shown in the inset of Figure 7(a), are decreased drastically by almost 60 ~ 70%, from 0.9 ~ 1.05 W m^−1^ K^−1^ for the *x* = 0 sample in the temperature range of 323 ~ 773 K to 0.35 ~ 0.45 W m^−1^ K^−1^ for the *x* = 0.08 sample. Moreover, the lattice component, *κ_l_*, is found to decrease gradually with *x*, optimally to 1.05 W m^−1^ K^−1^ at 323 K with *x* = 0.08 (in Figure 6(b)). This can be related to the improved phonon scattering due to the alloying effect with enhanced mass-fluctuation as well as the presence of Cu_3_SbS_3_, which also exhibits very low *κ_l_* of 0.2 ~ 0.3 W m^−1^ K^−1^ at 300 ~ 623 K [41] with a high lattice vibrational anharmonicity due to the 5s^2^ orbital lone-pair electrons of the trivalent Sb atoms. As temperature rises up to 673 K, the *κ_l_* values converge gradually to 0.3 ~ 0.4 W m^−1^ K^−1^ in the *x* = 0 sample. The ultralow *κ_l_* in CTS has been reported frequently, and can be caused by the complex microstructure with disordered cation arrangement, which can also be referred to other materials like TmAlB_4_ [42]. The convergence of *κ_l_* at high temperatures indicates the predominant role of the cation-disordered feature on phonon transport interruption.

**Figure 8.**
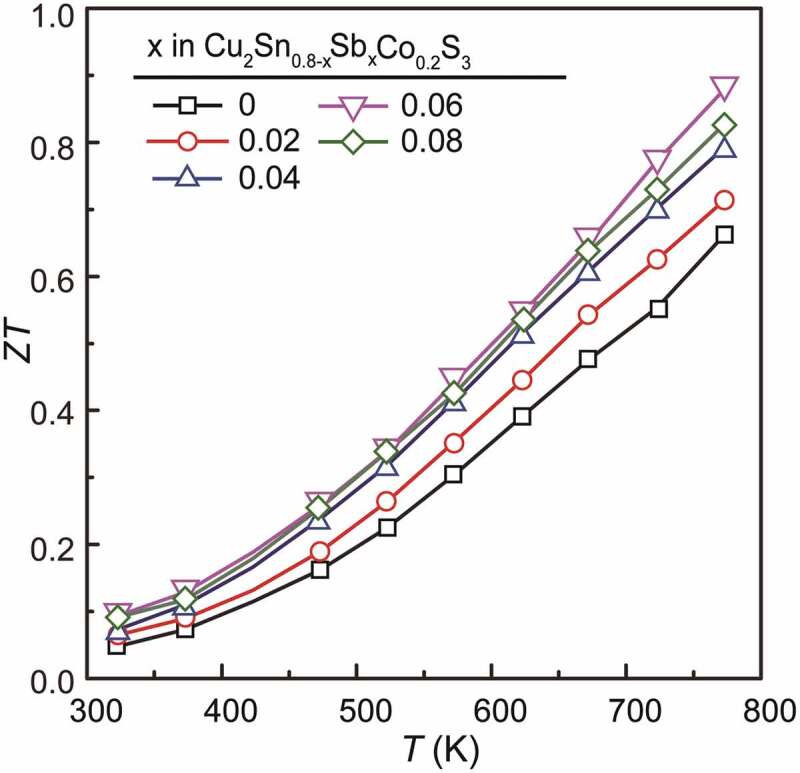
*ZT* values vs temperature for Cu_2_Sn_0.8-*x*_Sb*_x_*Co_0.2_S_3_ (*x* = 0, 0.02, 0.04, 0.06 and 0.08) samples. The peak value of 0.88 was obtained in the *x* = 0.06 sample at 773 K, which is 35% larger than that of *x* = 0 one, and is among the tops for CTS-based TE materials so far

Finally, the *ZT* value calculated using the obtained parameters are plotted in Figure 8. The *x* = 0 sample showed a maximal value of 0.65 at 773 K, which is smaller than the previous one [20], due to the higher *κ_e_* in the present sample which was sintered at a higher temperature for improved density. Thanks to the maintained *PF* and the suppressed *κ_e_* and *κ* after Sb substitution, the *ZT* values are improved effectively to about 0.88. We also compared the data of Cu_2_Sn_0.74_Co_0.2_Sb_0.06_S_3_ with other Cu-Sn-S ternary compounds in Table 3, including Cu_3_SnS_4_- [43], Cu_7_Sn_3_S_10_- [44,45] and Cu_7_Sn_4_S_16_- [46], Cu_5_Sn_2_S_7_- [37] based materials, indicating a prominent performance of our samples especially for the electrical transport properties.

**Table 3.** Electrical conductivity, Seebeck coefficient, power factor, thermal conductivity, lattice thermal conductivity, *ZT* and temperature for several high performance Cu-Sn-S TE materials at high temperature, including Cu_3_SnS_4_- [43], Cu_7_Sn_3_S_10_- [44,45], Cu_4_Sn_7_S_16_- [46], Cu_5_Sn_2_S_7_- [37] based materials and Cu_2_Sn_0.74_Co_0.2_Sb_0.06_S_3_ in this work

samples	*σ* (Scm^−1^)	*S* (μVK^−1^)	*PF* (μWcm^−1^K^−2^)	*κ* (Wm^−1^K^−1^)	*κ*_l_ (Wm^−1^K^−1^)		*ZT*	*T* (K)
Cu_3_Sn_1.2_S_4_	200	180	6.5	0.69		0.39	0.75	790
Cu_7_Sn_3_S_9.1_Cl_0.9_	158	212	7.1	0.66		0.43	0.8	750
Cu_7_Sn_3_S_9_Br	178	205	7.35	0.58		0.4	0.95	750
Cu_4_Sn_7.5_S_15_Se	26	294	2.24	0.39		0.35	0.5	890
Cu_2.075_Sn_0.925_S_3_	285	85	10.3	2.5		0.55	0.28	700
This work	390	165	10.4	0.94		0.41	0.88	773

## 4. Summary

The findings in this work demonstrate that, Sb-doping in the Co-doped CTS, can induce phase transformation from cubic to tetragonal structure, and serve as a donor and be used to tailor the hole concentration by charge compensation and incorporate the extra states of Sb in the VB, leading to a proper reduction of hole concentration while m* is enhanced. This gives rise to a maintained high *PF* of around 10.5 μW cm^−1^ K^−2^ at ~773 K and a simultaneously reduced *κ_e_*. In addition, *κ_l_* was further reduced due to the alloying effect and maintained at a low level of 0.3 ~ 0.4 W m^−1^ K^−1^ at ~723 K as a result of the lattice vibration nature of highly cation-disordered CTS. As a result, a high *ZT* of 0.88 has been realized at 773 K in the *x* = 0.06 sample (Cu_2_Sn_0.74_Sb_0.06_Co_0.2_S_3_), which stands among the tops so far of the CTS-based diamond-like ternary sulfides. Moreover, the preferential change in t-CTS component due to Sb doping suggests the possibility of further tailoring crystal chemistry, microstructural and electronic transport in the polymorphic CTS bulk by appropriate doping.
